# Bedside teaching: an invaluable tool in undergraduate medical education

**DOI:** 10.1192/bjo.2021.431

**Published:** 2021-06-18

**Authors:** Kenneth Ruddock

**Affiliations:** NHS Lanarkshire

## Abstract

**Aims:**

Bedside teaching is one of the most important modalities in medical education. Sir William Osler stated, “Medicine is learned by the bedside and not in the classroom”. Despite this, the use of bedside teaching in the undergraduate curriculum has been declining, potentially due to changes in course design, increasing clinical workloads and reducing inpatient numbers. In my role as a Clinical Teaching Fellow (CTF), I have aimed to maximise bedside teaching and promote it as the primary approach for student learning.

**Method:**

As a CTF, I deliver teaching to students from the Universities of Glasgow and Edinburgh during their placements in NHS Lanarkshire. Weekly teaching is provided to groups of 2-4 students, with around 50% of sessions delivered ‘at the bedside’.

Within psychiatry, there is a vast range of potential bedside teaching topics. Given the length of time required to conduct a full psychiatric history and mental state examination (MSE), teaching sessions instead focus on one specific component of the patient interview, for example, assessing perceptual abnormalities or delusions, conducting a substance use history or exploring social circumstances and the functional impact of illness. This approach allows for more focussed feedback and teaching. Session structure is based upon Cox's model of bedside teaching, which I have modified slightly for the psychiatry setting.

Student feedback has been collected via an anonymous electronic end-of-block questionnaire.

**Result:**

Qualitative feedback reveals that students in NHS Lanarkshire value bedside teaching, with one student describing it as “informative, comprehensive and relevant for upcoming exams and clinical practice”.

There are a number of potential barriers to consider when delivering bedside teaching in psychiatry. These include issues identifying suitable patients who can provide informed consent to participate and the ethical concerns regarding exploring difficult subjects such as suicide risk assessment with patients for purely educational purposes.

These issues can be overcome; in inpatient units, there is usually a small cohort of patients who are able to consent and engage in student teaching, and difficult subjects can alternatively be addressed during role-play or simulation sessions.

**Conclusion:**

Despite its challenges, bedside teaching can be an enjoyable and rewarding approach in undergraduate medical education, with feedback revealing it is positively received in NHS Lanarkshire. By utilising Cox's model and focussing on specific aspects of MSE and history-taking, bedside teaching is more accessible and an invaluable tool for psychiatric teaching. Clinicians and educators are encouraged to keep the patient at the centre of student learning.

